# Naloxegol to Prevent Constipation in ICU Adults Receiving Opioids: A Randomized Double-Blind Placebo-Controlled Pilot Trial

**DOI:** 10.1155/2022/7541378

**Published:** 2022-03-20

**Authors:** Matthew S. Duprey, Harmony Allison, Erik Garpestad, Andrew M. Riselli, Anthony Faugno, Eric Anketell, John W. Devlin

**Affiliations:** ^1^School of Public Health, Brown University, Providence, RI, USA; ^2^Division of Gastroenterology, Tufts Medical Center, Boston, MA, USA; ^3^Division of Pulmonary, Critical Care and Sleep Medicine, Tufts Medical Center, Boston, MA, USA; ^4^Department of Radiology and Biomedical Imaging, University of California San Francisco, San Francisco, CA, USA; ^5^Department of Nursing, Tufts Medical Center, 800 Washington Street, Boston, MA, USA; ^6^School of Pharmacy, Northeastern University, Boston, MA, USA

## Abstract

**Background:**

Constipation is frequent in critically ill adults receiving opioids. Naloxegol (N), a peripherally acting mu-receptor antagonist (PAMORA), may reduce constipation. The objective of this trial was to evaluate the efficacy and safety of N to prevent constipation in ICU adults receiving opioids. *Methods and Patients*. In this single-center, double-blind, randomized trial, adults admitted to a medical ICU receiving IV opioids (≥100 mcg fentanyl/day), and not having any of 17 exclusion criteria, were randomized to N (25 mg) or placebo (P) daily randomized to receive N (25mg) or placebo (P) and docusate 100 mg twice daily until ICU discharge, 10 days, or diarrhea (≥3 spontaneous bowel movement (SBM)/24 hours) or a serious adverse event related to study medication. A 4-step laxative protocol was initiated when there was no SBM ≥3 days.

**Results:**

Only 318 (20.6%) of the 1542 screened adults during the 1/17–10/19 enrolment period met all inclusion criteria. Of these, only 19/381 (4.9%) met all eligibility criteria. After 7 consent refusals, 12 patients were randomized. The study was stopped early due to enrolment futility. The N (*n* = 6) and *P* (*n* = 6) groups were similar. The time to first SBM (N 41.4 ± 31.7 vs. P 32.5 ± 25.4 hours, *P* = 0.56) was similar. The maximal daily abdominal pressure was significantly lower in the N group (N 10 ± 4 vs. P 13 ± 5, *P* = 0.002). The median (IQR) daily SOFA scores were higher in N (N 7 (4, 8) vs. P 4 (3, 5), *P* < 0.001). Laxative protocol use was similar (N 83.3% vs. P 66.6%; *P* = 0.51). Diarrhea prevalence was high but similar (N 66.6% vs. *P* 66.6%; *P* = 1.0). No patient experienced opioid withdrawal.

**Conclusions:**

Important recruitment challenges exist for ICU trials evaluating the use of PAMORAs for constipation prevention. Despite being underpowered, our results suggest time to first SBM with naloxegol, if different than P, may be small. The effect of naloxegol on abdominal pressure, SOFA, and the interaction between the two requires further research.

## 1. Introduction

Opioids are frequently administered in the intensive care unit (ICU) [1]. Constipation, the inability to pass stool for ≥3 consecutive days, is a frequent sequela of opioid use in this setting, and associated with nausea and vomiting, abdominal distension, longer mechanical ventilation, and reduced enteral feeding [2–6]. Management of gastrointestinal dysfunction during critical illness remains an important research area [7]. The use of ICU laxative protocols often fail to reduce constipation and do not improve nutrition tolerance or reduce mechanical ventilation [8–10]. Neither the osmotic nor stimulant laxatives used in protocols target the *µ*-opioid receptor [6–11]. Additionally, laxative use in may increase the risk for diarrhea [9]. While use of a daily laxative protocol is associated with improved organ function in mechanically ventilated adults [12], the association between constipation, abdominal pressure, and organ function remains unclear.

Naloxegol, a pegylated derivative of naloxone, is an oral peripherally acting antagonist (PAMORA) that antagonizes *µ*-opioid receptors in the enteric nervous system while preserving central nervous system (CNS)-mediated analgesia [4]. It is currently FDA-approved for the treatment of opioid-induced constipation (OIC) in adults with either cancer or noncancer pain [13, 14]. While the addition of methylnaltrexone, another PAMORA, has not been shown to be beneficial when added to a laxative protocol to *treat* opioid-induced constipation in the ICU [15], the use of a PAMORA to *prevent* opioid-induced constipation in the ICU has not been rigorously evaluated.

## 2. Aim

We designed a phase II randomized trial to assess the feasibility and clinical effects of naloxegol as a strategy to prevent constipation in critically ill adults receiving opioid therapy. We hypothesized naloxegol would prevent constipation, reduce laxative use, and lower abdominal pressure.

## 3. Materials and Methods

### 3.1. Study Design

This prospective, randomized, double-blind, placebo-controlled, pilot study was conducted in the 10-bed medical ICU at Tufts Medical Center (TMC), a 400-bed academic center in Boston, MA. At the time of the study, decisions regarding constipation prevention or treatment were left to individual clinicians. The study was approved by the TMC institutional review board (IRB#10243), and written informed consent was obtained from each subject before randomization. The trial was registered online prior to recruitment (NCT02977286).

### 3.2. Patient Population

Between January 2017 and October 2019, we enrolled consecutive eligible and consenting adults receiving scheduled opioid therapy ((≥100 mcg IV fentanyl (or equivalent) for ≥24 hours) who were expected to survive ≥48 hours. 27 exclusion criteria, all of which were derived from the naloxegol package insert (16) or through research team discussion, are listed in Table 1. Six months into the study, the TMC IRB agreed to remove the exclusion criteria precluding the enrolment of patients with end stage renal disease (ESRD) given new evidence to suggest naloxegol does not accumulate in ESRD [17].

### 3.3. Randomization

Eligible patients were randomly assigned to receive naloxegol or an identical placebo. Study assignments were generated by the investigational drug service (IDS) using computer-generated randomization blocks of 4. Subjects, patients, clinicians, and study personnel were blinded to study the drug assignment.

### 3.4. Study Interventions

Based on the package insert, naloxegol 25 mg or placebo was administered orally at 9am daily. A lower daily dose of 12.5 mg was administered on days of moderate CYP3A4 inhibitor use (e.g., diltiazem and fluconazole) or moderate renal insufficiency (creatinine clearance (CrCL) ≤ 60 mL/min) [16]. When oral administration was not feasible, naloxegol was crushed and administered via a feeding tube [16]. All subjects received docusate sodium 100 mg twice/day. Patients not having a spontaneous bowel movement (SBM) within three days of ICU scheduled opioid initiation were initiated on a 4-step study laxative protocol, developed through literature review and investigator consensus (Supplemental Table 1) [8–10]. The study laxative protocol was stopped when a SBM occurred.

The study drug was administered until the following: (1) excessive diarrhea (≥3 SBM with a Bliss Score of 3 or 4 in a 24-hour period) [18] for ≥48 hours (despite naloxegol being held in the prior 24 hours); (2) no SBM after 6 days therapy and level-4 laxative protocol use; (3) scheduled opioid therapy stopped ≥24 hours and ≥1 SBM; (4) ≥ 10 days of study drug, ICU discharge, or death (whichever occurred first); and (5) an adverse event potentially attributable to the study drug deemed severe enough to warrant discontinuation. All opioid therapy, enteral nutrition, mobility efforts, and spontaneous breathing trial efforts were managed at the ICU team's discretion.

### 3.5. Data Collection

Data were collected at enrolment and daily for up to 10 days or ICU discharge/death. Baseline data included age, body mass index (BMI), the Acute Physiologic and Chronic Health Evaluation (APACHE)-II score [19], the modified Sequential Organ Function Assessment (mSOFA) score [20], admission diagnosis, time from last SBM at enrolment, and opioid exposure 24 hours prior to randomization. Data were collected on each ICU day included SBM number, size, and consistency using the 4-point Bliss criteria [18], opioid exposure, laxative protocol use, mSOFA, nutrition goal/volume delivered, fluid balance, q4h pain score (VAS-10 or CPOT) [1], q4h Sedation Agitation Scale (SAS) assessment [21] and q12 h Intensive Care Delirium Screening Checklist assessment [22]. On days a patient had a urinary catheter, a bladder pressure transducer was inserted (Bard® Intra-abdominal Pressure Monitoring Device, Murray Hill, NJ) and abdominal pressure was measured every 8 hours using standard institutional policies [23, 24]. Patients were evaluated one hour before and two hours after each study dose using the Clinical Opioid Withdrawal Scale (COWS) [25]. After training, all ICU assessments were conducted by the bedside nurse.

### 3.6. Outcomes

The primary study outcome was the time to first SBM during the ICU admission after randomization. Secondary SBM-related outcomes included the time to first SBM during the ICU admission after ICU opioid initiation, the number of SBMs/ICU day, and each SBM size and consistency. Other secondary efficacy outcomes included daily laxative protocol use, daily maximal abdominal pressure (and the % of patients ever with a pressure ≥12 mmHg or ≥ 20 mmHg) [24–26], highest daily mSOFA score [20], the daily enteral nutrition volume administered and daily goal reached, daily fluid balance, daily maximum pain score, and ICU coma (SAS = -1/-2), and delirium (ICDSC ≥4) occurrence. Safety outcomes included ICU days with diarrhea, rectal tube use, and difference in pre-post dose COWS score [25].

### 3.7. Statistical Analysis

With no planned or published naloxegol (or another PAMORA) study to prevent ICU constipation existing, we relied on 3-day estimates of SBM failure from a RCT evaluating early ICU protocolized laxative use to estimate sample size [27]. Therefore, enrolment of 36 subjects (18/group) would yield 95% power using an alpha of 0.05 if the true estimated SBM occurrence rate by day 3 was 80% in the naloxegol group and 40% in the placebo group.

All patients were analyzed by their randomization group according to the intention-to-treat principle. Continuous variables were reported as mean ± SD or median (interquartile (IQR) range). To test the primary outcome, we constructed Kaplan–Meier curves for each group for the time to first SBM and compared the curves using a log-rank test. For outcomes reported as a percentage of the time study drug was administered, a percentage was first calculated for each subject; the median (IQR) was then reported for each group. A *P* value of up to 0.05 was considered significant. All statistical analyses were performed using SAS 9.4 (SAS Institute, Cary, NC).

## 4. Results

### 4.1. Enrolment and Baseline Characteristics

**Figure 1:**
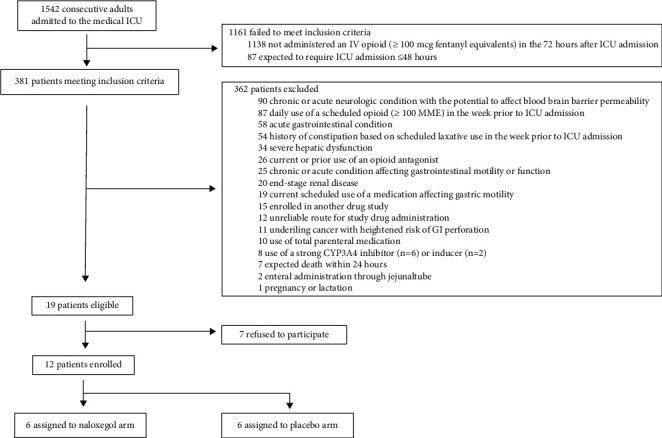
Patient screening, recruitment, and randomization. The number of patients excluded for each criterion sum is more than the total because some patients met more than one exclusion criteria.

From 1/2017–10/2019, we screened 1542 patients for eligibility; 1161 (75%) failed to meet both inclusion criteria (Figure 1). Among 381 remaining patients, 362 (95%) met one or more exclusion criteria (Table 1). Among the 362 excluded patients 101 (26.5%) had one exclusion criteria; 142 (39.2%) two, 44 (12.1%) three, and 75 (20.7%) ≥ 4. A chronic/acute neurologic condition (90 (24.9%)), pre-ICU scheduled opioid use ≥100 MME (87 (24.0%)), acute gastrointestinal disorder (58 (16.0%)), and pre-ICU scheduled laxative use (54 (14.9%)) were the most common exclusion criteria. Among the 19 patients eligible to enrol, 7 (37%) patients refused to participate leaving 12 randomized patients and who all received ≥1 study dose. The study was stopped early by the sponsor due to enrolment futility.

Patients randomized to naloxegol (*n* = 6) and placebo (*n* = 6) were similar at baseline (Table 2) and enroled, on average, 2 days after ICU admission. The median (IQR) ICU opioid exposure (in fentanyl equivalents) in the 24 hours prior to randomization far exceeded the inclusion criteria of 100 mcg fentanyl equivalents (naloxegol 1421 (650, 3538) vs. placebo 1600 (1104, 2381) mcg).

### 4.2. Drug Dosing and Spontaneous Bowel Movements

**Figure 2:**
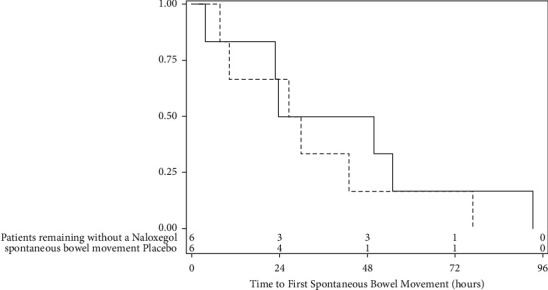
Time to first spontaneous bowel movement from the time of enrolment. The Kaplan–Meier curve for the time to the first spontaneous bowel movement occurrence between the naloxegol and placebo groups during the ICU stay from the time of enrolment (log rank *P* value = 0.56). The naloxegol and placebo lines at the bottom of the figure refer to the patients still receiving study drug.

The median (IRQ) duration of study drug administration (naloxegol 1.6 (1.5, 2.4) vs placebo 2.5 (1.6, 2, 2); *P* = 0.69), daily dose (naloxegol 18 (11, 23) mg vs placebo 21 (14, 24) mg; *P* = 0.9), and weight-based daily dose (per actual body weight) (naloxegol 0.17 (0.10, 0.21) mg/kg vs placebo 0.21 (0.14, 0.24) mg/kg; *P* = 0.73). The time to the first SBM after study enrolment was not different between the naloxegol and placebo groups (Figure 2); occurring at 41.4 ± 31.7 hours in the naloxegol group and at 32.5 ± 25.4 hours in the placebo group (*P* = 0.56) (Table 3). Additionally, the time to the first SBM was not different between the two groups from the time opioids were initiated to in the ICU (Supplemental Figure 1) or the time of the last documented SBM before enrolment (Supplemental Figure 2). The size and consistency of the first SBM was not different between the two groups (Table 3). The total daily number of SBMs in the naloxegol and placebo groups stratified by study laxative protocol use is presented in Supplemental Figure 3.

### 4.3. Abdominal Pressure and Organ Function

Abdominal pressure scores were collected from 6 (100%) patients in the naloxegol group (71 assessments) and 4 (66%) patients in the placebo group (99 assessments). For one placebo patient, the bladder pressure transducer was not in stock at the time of enrolment and in the other the bladder catheter was removed on first study day (as per the ICU catheter-associated urinary tract infection protocol). The average daily maximum abdominal pressure score in the first 7 days from enrolment was significantly lower in the naloxegol group (10.0 vs. 13.4 mmHg, *P* = 0.002) (Table 3). During this same period, fewer patients in the naloxegol group had scores ≥12 mmHg (*P* = 0.003) while scores ≥20 mmHg were numerically greater but not statistically different (*P* = 0.12). However, the average daily abdominal pressures between groups was not different (*P* = 0.11) (Supplemental Figure 4). The median maximal daily mSOFA score was notably higher among individuals randomized to naloxegol (*P* < 0.001).

### 4.4. Secondary Clinical Outcomes

Overall laxative protocol use was not different between the two groups (*P* > 0.99); although no naloxegol and 2 placebo patients required laxative protocol Step 3 or 4 (Table 3). Daily opioid and propofol use (the only continuous sedative used in the study) were similar between groups. Neither the daily nutrition goal or the % of the goal reached was different. Maximal pain scores and days without coma or delirium or without mechanical ventilation were also not different.

### 4.5. Safety

Four patients in each group had ≥1 diarrhea episode; time to this first episode was more than twice as long in the placebo group (Table 4). Within each group, diarrhea resolved within 24 hours in two patients and persisted ≥48 hours in two. Three naloxegol, but only 1 placebo patient, required a rectal tube. Preadministration and postadministration COWS score were similar in both groups.

## 5. Discussion

Our pilot study is the first published randomized, double-blind, placebo-controlled trial evaluating a PAMORA for the prevention of opioid constipation in the ICU. Clinicians should extrapolate our results with caution given our trial was stopped early by the sponsor; only one-third of our planned enrolment target was able to be reached. One should not conclude from our trial naloxegol does not improve SBM occurrence nor change the way they prevent/treat opioid constipation until larger trials are conducted. The study protocol we developed, the recruitment challenges we experienced, and the results we present for the 12 enrolled patients will help inform future ICU PAMORA investigations.

In our protocol, we tried to define an ICU daily opioid dose (≥100 mcg fentanyl equivalents) that would be associated with a greater likelihood for opioid-associated constipation. However, opioid prescribing practices during the 2017–2019 study period were surprisingly low. American (compared to European) trials have not found analgosedation improves outcome [28]. Opioid-induced coma is associated with longer ventilation and greater mortality [29] and ICU opioid use is associated with greater delirium [30]. In the face of the ongoing opioid epidemic, clinicians are prescribing fewer opioids and more non-opioid analgesics [31–33]. The study ICU has participated in numerous sedation trials all of which are focused on light sedation and sedative (vs opioid) use [34, 35]. To this end, only 26% of the 1542 screened patients received fentanyl ≥100 mcg on ≥ 1 ICU day. If opioid prescribing practices at our center are similar elsewhere, outside of the current COVID-19 pandemic, future ICU PAMORAs studies may be challenging to conduct.

In our study, we focused on excluding patients taking higher-dose opioid therapy prior ICU admission or who had a history of moderate-severe constipation. However, among the 381 patients who met all study inclusion criteria, 254 had one or both these exclusion criteria. Future research is required regarding the use of PAMORAs in critically ill adults with a history of substantial pre-ICU opioid, constipation, or with active gastrointestinal conditions. Although the risk for naloxegol CNS penetration and opioid withdrawal reactions may be greater in critically ill adults, particularly those with chronic or acute neurological conditions [13], more research to clarify this potential risk in the ICU is important.

Our approach to delaying the initiation of a gradated ICU laxative protocol until 3 days after randomization may not represent standard practice. While SBM frequency remains a clinically relevant ICU outcome, our focus on evaluating naloxegol to prevent constipation (i.e., shorten the time to the first SBM) may not be the most relevant endpoint in the ICU despite many Rome IV opioid-induced constipation criteria (e.g., straining or manual maneuvers during defection) being hard to measure [36]. Future PAMORA studies should use an ICU laxative protocol earlier and evaluating ICU days spent without a SBM, like many ICU coma and delirium trials [35], rather than the time to the first SBM. The lower average intra-abdominal pressure scores in the naloxegol group highlights the importance of further evaluating this outcome in future PAMORA trials. Intra-abdominal hypertension, and the potential organ dysfunction associated with it, remains important clinical outcomes in critically ill adults [36]. In the naloxegol group, it remains unclear how the intra-abdominal pressure scores were lower yet the average SOFA score was higher. Newer approaches to evaluate ICU organ dysfunction over time in should be considered in future trials [37]. The results of our study suggest naloxegol appears to be safe, although diarrhea, if it occurs, may happen faster and be more severe with naloxegol. Opioid withdrawal was not detected in any patient receiving naloxegol.

Our study has important limitations. Its small sample size and the fact only one-third of the planned enrolment patients was reached and makes it underpowered to detect a difference in the primary outcome. Our strict enrolment criteria limit its external validity. The study took place at only center, patients and clinical practices may be different at other centers. Our results may not apply to surgical patients. Although baseline characteristics were not statistically different, including patient medical and surgical history, the groups may not have been evenly matched. Our analysis did not account for other potentially influencing factors on gastrointestinal function, including medications (e.g., sorbitol content) and degree of mobilization. Despite the limitations of our study, and the gaps in knowledge that remain regarding PAMORA use in the ICU, initiation of a PAMORA-like naloxegol in patients with severe opioid-associated constipation who are resistant to multicomponent laxative protocol use appears to be a safe and reasonable therapeutic approach.

## 6. Conclusions

Important recruitment challenges exist for trials evaluating PAMORAs for ICU constipation prevention. Our results suggest the time to first SBM with naloxegol use, if different than placebo, may be small. The effect of naloxegol on abdominal pressure, SOFA scores, and the interaction between the two, requires further research. Naloxegol may be associated with an earlier occurrence of more severe diarrhea. Future studies should evaluate patients with pre-ICU opioid use and constipation, better control for laxative protocol use, and carefully evaluate abdominal pressure and organ dysfunction.

## Figures and Tables

**Table 1:** Study exclusion criteria.


Daily use of a scheduled opioid (≥100 MME) or methadone at any dose in the week prior to ICU admission
History of constipation as defined by the scheduled use of bisacodyl, senna, lactulose, PEG 3350 (MiraLAX®), and/or saline enema (Fleet® saline enema) prior to ICU admission
Current scheduled use of a medication affecting gastric motility (e.g., metoclopramide, domperidone, erythromycin, and loperamide)
Acute GI condition (e.g., clinical evidence of acute fecal impaction or complete obstruction, acute surgical abdomen, and acute GI bleeding)
Chronic or acute condition affecting GI motility or function (e.g., inflammatory bowel disease requiring immunosuppressive therapy, symptomatic clostridium difficile, active diverticular disease, and surgery on the colon or abdomen within 60 days of ICU admission)
Current or previous use of an opioid antagonist agent (e.g., naloxegol and methylnaltrexone) in the past 30 days
Current use of total parenteral nutrition
Current use of a medication known to be a strong CYP3A4 inhibitor (e.g., itraconazole, ketoconazole, voriconazole, lopinavir/ritonavir, indinavir/ritonavir, ritonavir, clarithromycin, nefazodone)
Current use of a medication known to be a strong CYP3A4 inducer (e.g., rifampin, carbamazepine, St. John's wort)
Known serious or severe hypersensitivity to Movantik® (naloxegol) or any of its excipients
Severe hepatic dysfunction, defined as (i) INR ≥2.0 (not related to warfarin therapy) and total bilirubin ≥2 or (ii) diagnosis of liver cirrhosis defined by child-pugh class B or C, or (iii) acute liver disease is the primary reason for current ICU admission
Chronic or acute neurologic condition that may affect the permeability of the blood-brain barrier (e.g., multiple sclerosis, recent brain injury, Alzheimer's disease, uncontrolled epilepsy, acute stroke, and acute meningitis)
Underlying cancer associated with heightened risk of GI perforation (e.g., underlying malignancies of the GI tract or peritoneum, recurrent or advanced ovarian cancer, and vascular endothelial growth factor inhibitor treatment)
Administration of enteral nutrition through a jejunal tube
Unreliable method for enteral, gastric, or oral medication administration (e.g., no feeding tube and NG tube on suction)
Inability to enrol and initiate study medication within 72 hours of first initiating IV opioid therapy in the ICU
Patients expected to expire within 24 hours
Pregnant or actively lactating females
Current participation in another interventional clinical study
Inability to obtain informed consent from either the patient or their legally authorized representative
Medical ICU or attending physician objection to patient enrolment

CYP3A4: cytochrome P450 3A4; GI: gastrointestinal; ICU: intensive care unit; IV: intravenous; INR: international normalized ratio; MME: morphine milligram equivalents; PEG: polyethylene glycol; NG: nasogastric.

**Table 2:** Patient characteristics at baseline.

Variable	Naloxegol, *n* = 6	Placebo, *n* = 6
Age, years, mean ± SD	51 ± 23	64 ± 11
Male, N (%)	3 (50)	2 (33)
BMI, mean ± SD	40 ± 13	35 ± 16
Apache-II score, mean ± SD	20 ± 6	19 ± 7
SOFA score, mean ± SD	8 ± 4	6 ± 2
Medical (vs. surgical), N (%)	6 (100)	6 (100)
Mechanically ventilated, N (%)	6 (100)	6 (100)
Hours in the ICU before enrolment, mean ± SD	50 ± 21	44 ± 21
Admission diagnosis, N (%)		
Pneumonia	2 (33)	2 (33)
Cardiac	2 (33)	2 (33)
Respiratory failure	1 (17)	1 (17)
ARDS	1 (17)	1 (17)
Last SBM prior to enrolment, days, mean ± SD	3 ± 2	3 ± 2
Scheduled/continuous IV opioid medication, *n* (%)		
Fentanyl	5 (83)	6 (100)
Hydromorphone	1 (17)	0 (0)
Opioid exposure in the prior 24 hours		
Total IV fentanyl equivalents (mcg), median (IQR)	1420 (650, 3548)	1600 (1104, 2381)
IV fentanyl equivalents mcg/kg/hr, median (IQR)	0.54 (0.25, 0.98)	0.61 (0.29, 0.94)
Continuous propofol use in the prior 24 hours		
N (%)	4 (66)	4 (66)
Infusion rate, mcg/kg/min, median (IQR)	32 (25, 37)	35 (27, 43)
Location prior to ICU admission, *n* (%)		
Emergency department	1 (17)	1 (17)
Hospital ward	1 (17)	2 (33)
ICU at outside hospital	3 (50)	2 (33)
Ward at outside hospital	1 (17)	1 (17)
Past medical history, *n (%)*		
Asthma/COPD	1 (17)	0 (0)
Diabetes	2 (33)	1 (17)
GERD	2 (33)	1 (17)
Heart failure	0 (0)	0 (0)
Hypertension	2 (33)	3 (50)
Past surgical history, *n (%)*		
Abdominal	0 (0)	0 (0)
Cardiovascular	0 (0)	1 (17)
Orthopedic	1 (17)	2 (33)
Thoracic	0 (0)	0 (0)

ARDS: acute respiratory distress syndrome; APACHE: acute physiologic and chronic health evaluation; BMI: body mass index; COPD: chronic obstructive pulmonary disease; GERD: gastroesophageal reflux disease; ICU: intensive care unit; IQR: interquartile range; IV: intravenous; SBM: spontaneous bowel movement; SD: standard deviation; SOFA: sequential organ failure assessment.

**Table 3:** Clinical outcomes^A^.

Variable	Naloxegol, *n* = 6	Placebo, *n* = 6	*P* value
First SBM after enrolment			
Time to event, hours, mean ± SD	41 ± 32	33 ± 25	0.56
Size, N (%)			0.19
Small	1 (17)	4 (67)	
Medium	3 (50)	2 (33)	
Large	2 (33)	0 (0)	
Consistency, N (%)			0.77
Hard and formed	1 (17)	0 (0)	
Soft but formed	0 (0)	0 (0)	
Loose and unformed	4 (67)	4 (67)	
Liquid	1 (17)	2 (33)	
Maximum daily abdominal pressure score, mmHg, mean ± SD	10 ± 4	13 ± 5	0.002
Score ≥12 mmHg, N (%)	8 (15)	23 (31)	0.003
Score ≥20 mmHg, N (%)	0 (0)	4 (7)	0.12
Maximum daily SOFA score, median (IQR)	7 (4, 8)	4 (3, 5)	<0.001
Study laxative protocol			>0.99
Any use, N (%)	5 (83)	4 (67)	0.13
Highest level needed, N (%)			
Step 1	1 (20)	1 (25)	
Step 2	4 (80)	1 (25)	
Step 3	0	1 (25)	
Step 4	0	1 (25)	0.81
Total proportion of study days used, N/total (%)	11/54 (20)	9/51 (18)	
Days of scheduled/continuous IV fentanyl use, median (IQR)	5 (3, 7)	4 (3, 6)	0.77
Average daily IV fentanyl equivalents mcg/kg/hr, median (IQR)	0.44 (0.19, 0.72)	0.51 (0.24, 0.82)	0.84
Continuous propofol use			
N (%) ever during study	4 (66)	4 (66)	1.0
Days of propofol use, median (IQR)	5 (3–6)	4 (2–5)	0.73
Average daily infusion rate, mcg/kg/min, median (IQR)	28 (21, 33)	29 (20, 32)	0.81
Enteral nutrition			
Daily volume, mL, median (IQR)	103 (0, 240)	200 (0, 344)	0.06
Percent of daily goals met, mean ± SD	54	51	0.52
Daily fluid balance, mL, median (IQR)	−338 (−747, −102)	−210 (−660, −208)	0.22
Daily maximum pain score, median (IQR)	0 (0, 3)	0 (0, 0)	0.26
Days without coma or delirium, median (IQR)	1 (0.3, 2)	3 (2, 5)	0.20
Without coma, median (IQR)	3 (1, 3)	7 (4, 7)	0.17
Without delirium, median (IQR)	5 (4, 6)	6 (4, 7)	0.81
Days without mechanical ventilation, median (IQR)	0.5 (0.2, 5)	1 (0.3, 3)	0.69
Duration of ICU stay (days), median (IQR)	15 (11, 20)	10 (9, 14)	0.47
Reintubation during initial ICU stay, N (%)	1 (17)	2 (33)	>0.99

^A^Daily variable. Percentages are based on the individual days accrued by patients in each group. ICU: intensive care unit; IQR: interquartile range; mL: milliliters; N: number; SBM: spontaneous bowel movement; SD: standard deviation; SOFA: sequential organ function score.

**Table 4:** Safety outcomes.

Variable	Naloxegol, *n* = 6	Placebo, *n* = 6	*P* value
Patients with ≥1 episode of diarrhea, N (%)	4 (67)	4 (67)	>0.99
Time to first episode, hours, median (IQR)	40 (19, 66)	109 (48, 169)	0.57
Resolution of diarrhea within 24 hours after holding study drug and laxative protocol is stopped, N (%)	2 (50)	2 (50)	>0.99
Persistence of diarrhea ≥48 hours after study drug is held and laxative protocol is stopped, N (%)	2 (50)	2 (50)	>0.99
Use of a rectal tube, N (%)	3 (75)	1 (25)	0.54
Clinical opioid withdrawal scale	−0.1 ± 1.3	+0.2 ± 1.3	0.31
Difference in predose and postdose scores, mean ± SD			

IQR: interquartile range; N: number; SD: standard deviation.

## Data Availability

The data used to support the findings of this study are available from the corresponding author upon reasonable request.

## Supplementary Materials

Supplementary Materials
*Supplemental Figure 1*. Time to first spontaneous bowel movement from initiation of opioid in the ICU (the Kaplan–Meier curve for the time to the first spontaneous bowel movement occurrence between the naloxegol and placebo groups during the ICU stay from the time an opioid was first administered in the ICU (log rank *P* value = 0.62). The numbers in line with naloxegol and placebo at the bottom of the figure refer to the number of patients still receiving study treatment who had not experienced a spontaneous bowel movement. *Supplemental Figure 2.* Time to first spontaneous bowel movement from last documented known spontaneous bowel movement (the Kaplan–Meier curve for the time to the first spontaneous bowl movement occurrence between the naloxegol and placebo groups during the ICU stay from the last documented known spontaneous bowel movement either during (or before) the ICU admission (log rank *P* value = 0.98). The numbers in line with naloxegol and placebo at the bottom of the figure refer to the number of patients still receiving study treatment who had not experienced a spontaneous bowel movement. SBM = spontaneous bowel movement.) *Supplemental Figure 3.* Daily number of spontaneous bowel movements. (Daily number of SBM between naloxegol and placebo groups over the first 7 study days stratified by study laxative protocol use.) *Supplemental Figure 4*. Mean daily maximum abdominal pressure (Comparison of average daily abdominal pressure between naloxegol and placebo groups over the first 7 study days. (*P* value = 0.11)) *Supplemental Table 1*. Study laxative protocol.
