# Supplementary material for: Naloxegol to Prevent Constipation in ICU Adults Receiving Opioids: A Randomized Double-Blind Placebo-Controlled Pilot Trial
Source: Crit Care Res Pract. 2022 Mar 20;2022:7541378. doi: 10.1155/2022/7541378 (PMC8958087; doi:10.1155/2022/7541378)
Supplement: Supplementary Materials — Supplemental Figure 1. Time to first spontaneous bowel movement from initiation of opioid in the ICU (the Kaplan–Meier curve for the time to the first spontaneous bowel movement occurrence between the naloxegol and placebo groups during the ICU stay from the time an opioid was first administered in the ICU (log rank P value = 0.62). The numbers in line with naloxegol and placebo at the bottom of the figure refer to the number of patients still receiving study treatment who had not experienced a spontaneous bowel movement. Supplemental Figure 2. Time to first spontaneous bowel movement from last documented known spontaneous bowel movement (the Kaplan–Meier curve for the time to the first spontaneous bowl movement occurrence between the naloxegol and placebo groups during the ICU stay from the last documented known spontaneous bowel movement either during (or before) the ICU admission (log rank P value = 0.98). The numbers in line with naloxegol and placebo at the bottom of the figure refer to the number of patients still receiving study treatment who had not experienced a spontaneous bowel movement. SBM = spontaneous bowel movement.) Supplemental Figure 3. Daily number of spontaneous bowel movements. (Daily number of SBM between naloxegol and placebo groups over the first 7 study days stratified by study laxative protocol use.) Supplemental Figure 4. Mean daily maximum abdominal pressure (Comparison of average daily abdominal pressure between naloxegol and placebo groups over the first 7 study days. (P value = 0.11)) Supplemental Table 1. Study laxative protocol. [file 7541378.f1.pdf]

Appendix X. This appendix was part of the submitted manuscript and has been peer reviewed. It is posted as supplied by the authors

Supplemental Figures

Supplemental Figure 1. Time to first spontaneous bowel movement from initiation of opioid in the ICU

Kaplan-Meier curve for the time to the first spontaneous bowel movement occurrence between the naloxegol and placebo groups during the ICU stay from the time an opioid was first administered in the ICU (log rank *P* value = 0.62). The numbers in line with naloxegol and placebo at the bottom of the figure refer to the number of patients still receiving study treatment who had not experienced a spontaneous bowel movement.

Supplemental Figure 2. Time to first spontaneous bowel movement from last documented known spontaneous bowel movement

Kaplan-Meier curve for the time to the first spontaneous bowl movement occurrence between the naloxegol and placebo groups during the ICU stay from the last documented known spontaneous bowel movement either during (or before) the ICU admission (log rank *P* value = 0.98). The numbers in line with naloxegol and placebo at the bottom of the figure refer to the number of patients still receiving study treatment who had not experienced a spontaneous bowel movement. SBM = spontaneous bowel movement

Supplemental Figure 3. Daily number of spontaneous bowel movements

Daily number of SBM between naloxegol and placebo groups over the first 7 study days stratified by study laxative protocol use.

Supplemental Figure 4. Mean daily maximum abdominal pressure

Comparison of average daily abdominal pressure between naloxegol and placebo groups over the first 7 study days. (*P*-value = 0.11)

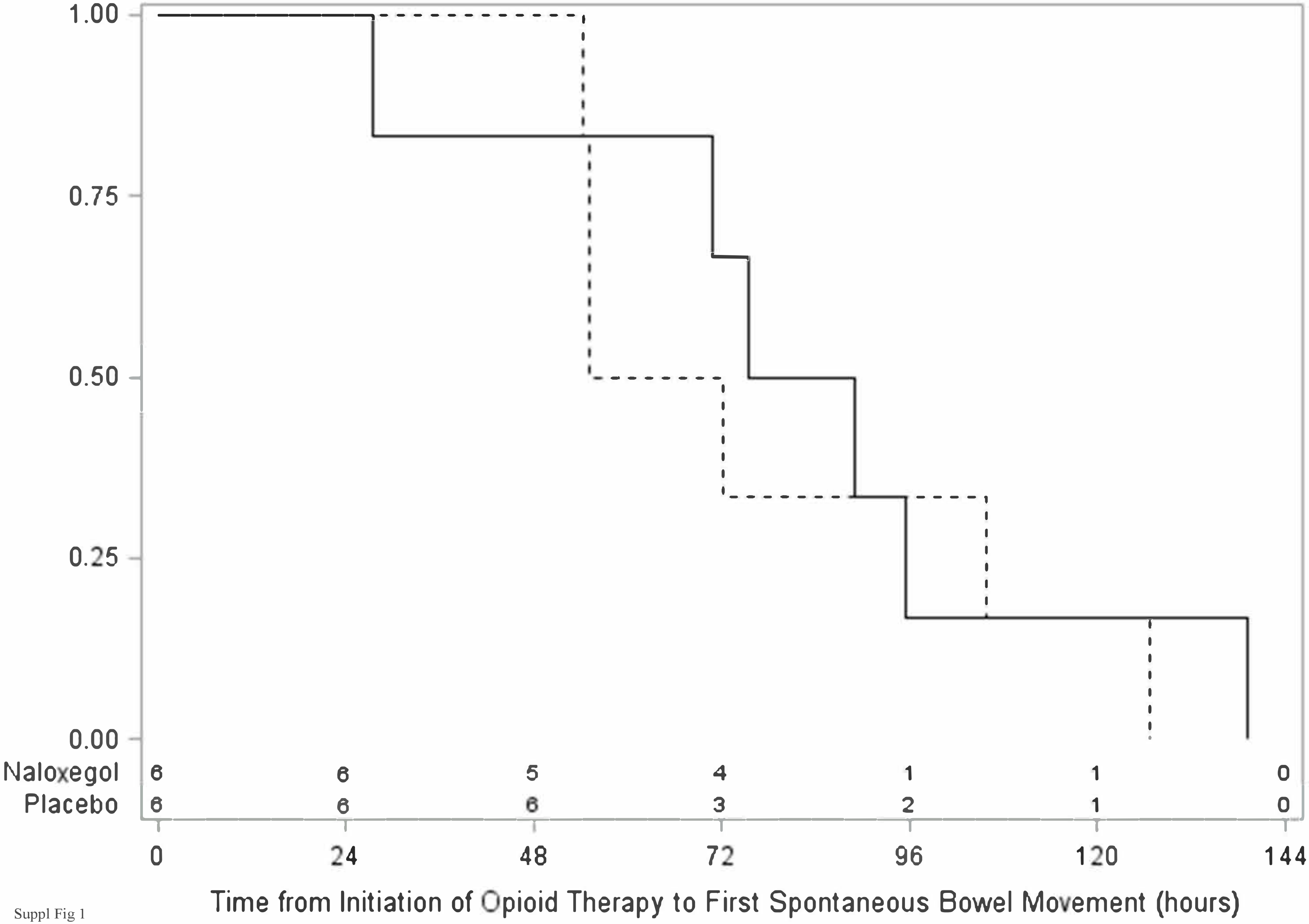
1.00
0.75
0.50
0.25
0.00
Naloxegol 6 6 5 4 1 1 0
Placebo 6 6 6 3 2 1 0
0
24
48
72
96
120
144
Time from Initiation of Opioid Therapy to First Spontaneous Bowel Movement (hours)

Suppl Fig 1

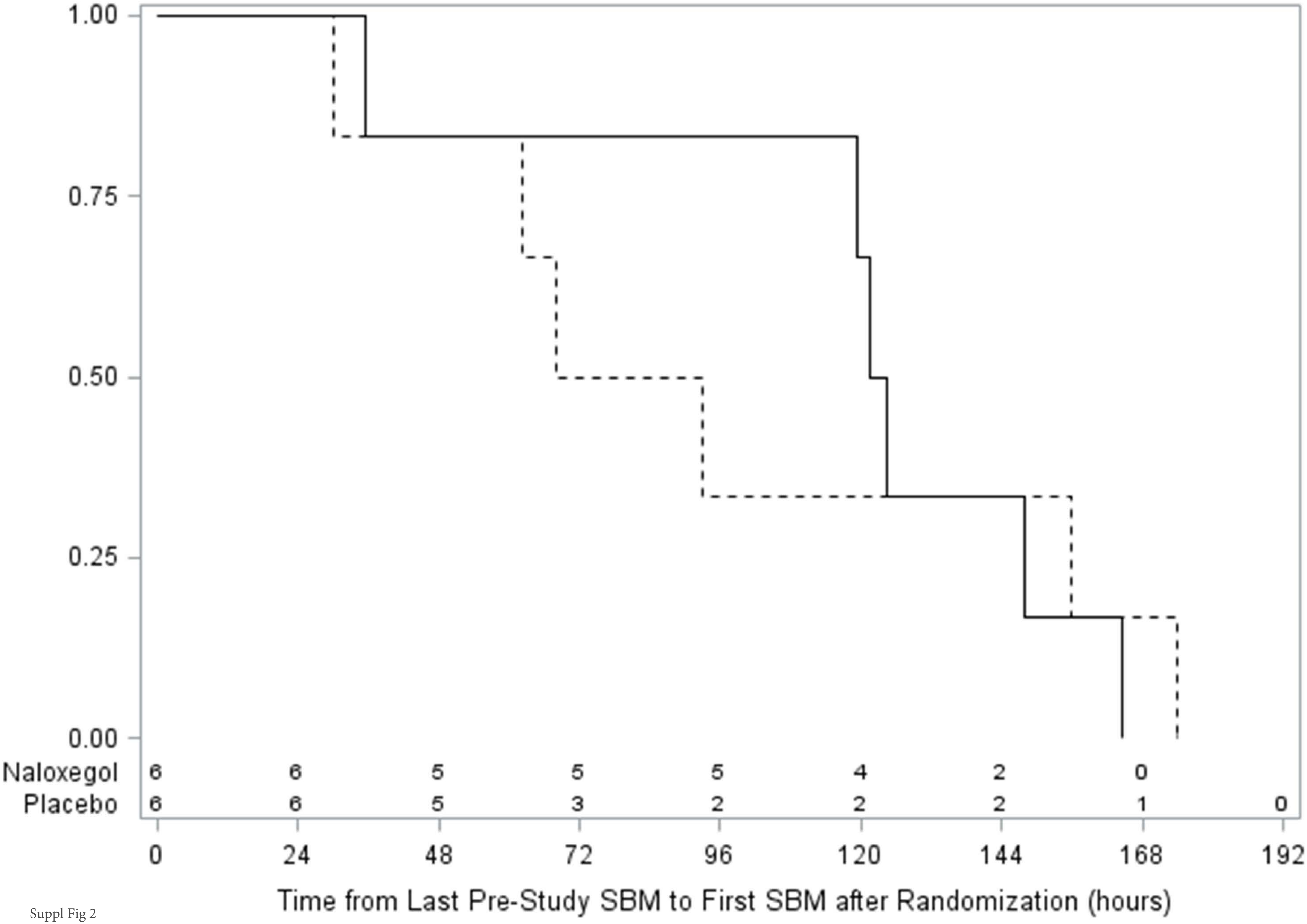

1.00
0.75
0.50
0.25
0.00
Naloxegol 6 6 5 5 5 4 2 0
Placebo 6 6 5 3 2 2 2 1 0
0 24 48 72 96 120 144 168 192
Time from Last Pre-Study SBM to First SBM after Randomization (hours)

Suppl Fig 2

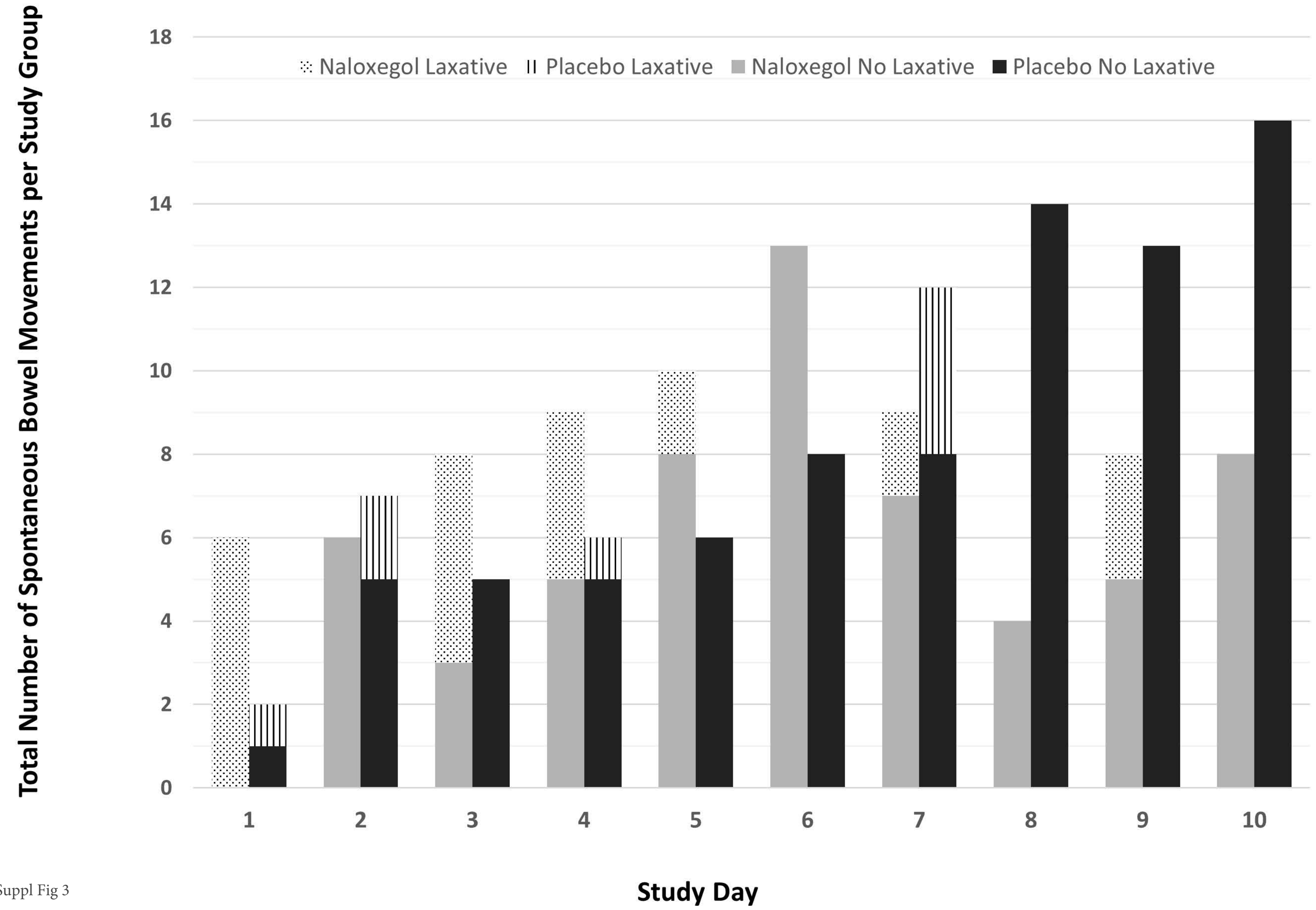
Naloxegol Laxative
Placebo Laxative
Naloxegol No Laxative
Placebo No Laxative
Total Number of Spontaneous Bowel Movements per Study Group
0
2
4
6
8
10
12
14
16
18
1
2
3
4
5
6
7
8
9
10
Study Day

Suppl Fig 3

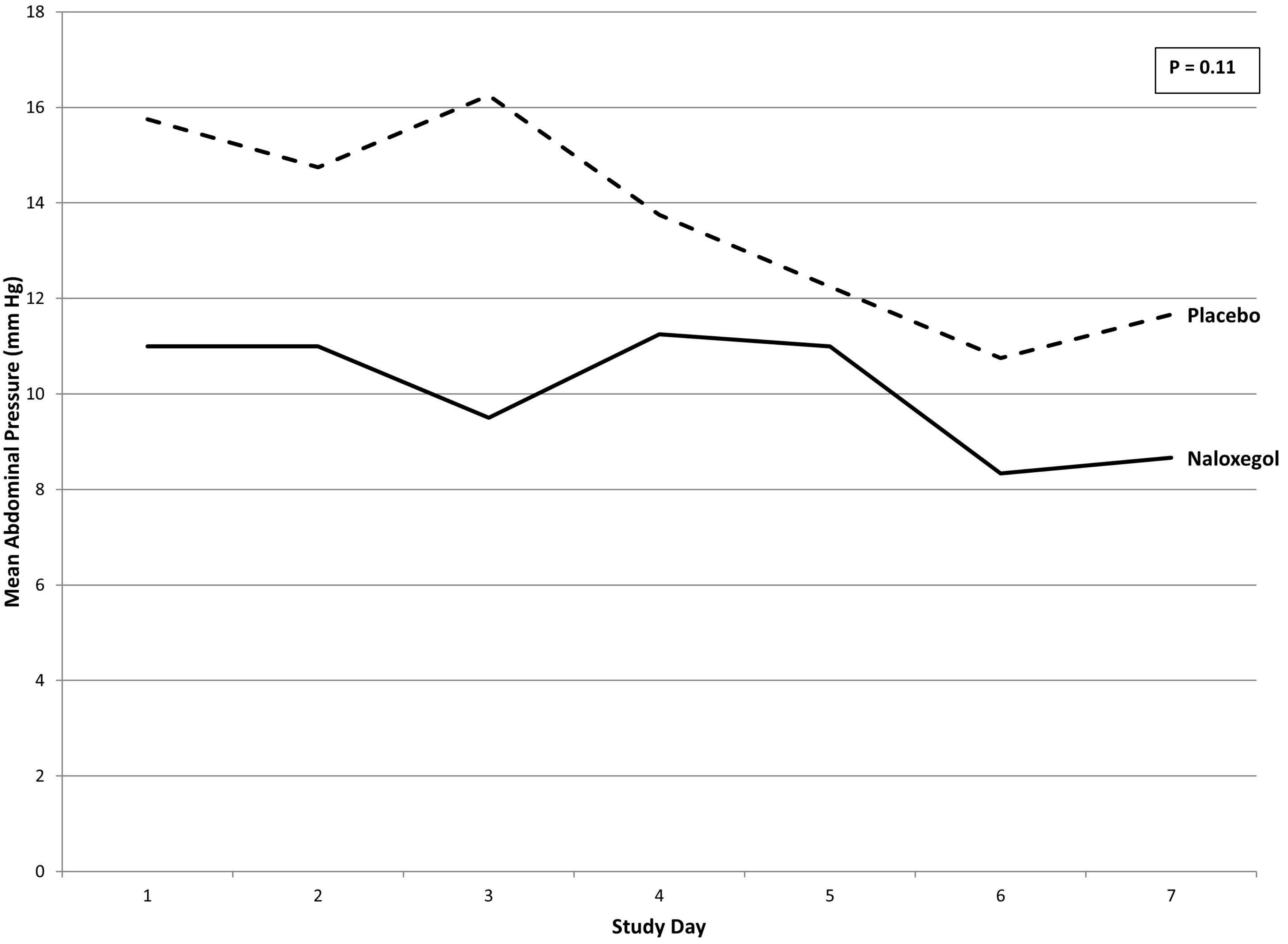
P = 0.11
Mean Abdominal Pressure (mm Hg)
18
16
14
12
10
8
6
4
2
0
Placebo
Naloxegol
1
2
3
4
5
6
7
Study Day

Suppl Fig 4

Supplemental Table 1. Study laxative protocol

*No spontaneous bowel movement in ≥ **3 days**[A]**?***

Step 1: Initiate: Senna one tablet (8.6 mg) PO/NG daily AND polyethylene glycol 3350 17 g PO/NG daily. If SBM occurs then stop step 1 therapy but keep patient on docusate.

*No spontaneous bowel movement in ≥ **4 days**[A]**?***

Step 2: Increase Senna to 2 tablets (17.2 mg) PO/NG daily AND polyethylene glycol 3350 to 34 g PO/NG daily and insert a bisacodyl suppository 10mg PR x 1. If bowel movement occurs then stop all laxative study protocol therapy but keep patient on docusate.

*No spontaneous bowel movement in ≥ **5 days**[A]?*

Step 3: Repeat Step 2 AND if no spontaneous bowel movement within 2 hours of administering the bisacodyl suppository, administer a 10oz bottle of magnesium citrate sodium phosphate. If bowel movement occurs then stop all study laxative protocol but keep patient on docusate.

*No spontaneous bowel movement in ≥ **6 days**[A]**?***

Step 4: **Discontinue study medication (but do not unblind patient assignment).** Repeat Step 3, initiate methylnatrexone (Relistor) sc x once and consider a consultation to gastroenterology or surgery. [Note: If patient weight is 38-62 kg then methylnaltrexone dose = 8mg; if patient weight is 62-144 kg then methylnaltrexone dose = 12mg].

[A]From the day of initiation of scheduled opioid therapy (ie. IV fentanyl ≥ 100mcg/day or equivalent)

CONSORT 2010 checklist of information to include when reporting a randomised trial*

Section/Topic	Item No	Checklist item	Reported on page No
**Title and abstract**			
	1a	Identification as a randomised trial in the title	Page 1
	1b	Structured summary of trial design, methods, results, and conclusions (for specific guidance see CONSORT for abstracts)	Page 3
**Introduction**			
Background and objectives	2a	Scientific background and explanation of rationale	Page 4
	2b	Specific objectives or hypotheses	Page 4
**Methods**			
Trial design	3a	Description of trial design (such as parallel, factorial) including allocation ratio	Page 4-5
	3b	Important changes to methods after trial commencement (such as eligibility criteria), with reasons	Page 5
Participants	4a	Eligibility criteria for participants	Page 5 and 17
	4b	Settings and locations where the data were collected	Page 4-5
Interventions	5	The interventions for each group with sufficient details to allow replication, including how and when they were actually administered	Page 6
Outcomes	6a	Completely defined pre-specified primary and secondary outcome measures, including how and when they were assessed	Page 6 and 7
	6b	Any changes to trial outcomes after the trial commenced, with reasons	Page 7
Sample size	7a	How sample size was determined	Page 7
	7b	When applicable, explanation of any interim analyses and stopping guidelines	N/A
Randomisation:			
Sequence generation	8a	Method used to generate the random allocation sequence	Page 5
	8b	Type of randomisation; details of any restriction (such as blocking and block size)	Page 5
Allocation concealment mechanism	9	Mechanism used to implement the random allocation sequence (such as sequentially numbered containers), describing any steps taken to conceal the sequence until interventions were assigned	Page 5
Implementation	10	Who generated the random allocation sequence, who enrolled participants, and who assigned participants to interventions	Page 5

Blinding	11a	If done, who was blinded after assignment to interventions (for example, participants, care providers, those assessing outcomes) and how	Page 5
	11b	If relevant, description of the similarity of interventions	N/A
Statistical methods	12a	Statistical methods used to compare groups for primary and secondary outcomes	Page 6
	12b	Methods for additional analyses, such as subgroup analyses and adjusted analyses	Page 6
**Results**			
Participant flow (a diagram is strongly recommended)	13a	For each group, the numbers of participants who were randomly assigned, received intended treatment, and were analysed for the primary outcome	
	13b	For each group, losses and exclusions after randomisation, together with reasons	Page 7-8 + Figure 1 + Table 1
Recruitment	14a	Dates defining the periods of recruitment and follow-up	Page 7
	14b	Why the trial ended or was stopped	Page 7
Baseline data	15	A table showing baseline demographic and clinical characteristics for each group	Table 2
Numbers analysed	16	For each group, number of participants (denominator) included in each analysis and whether the analysis was by original assigned groups	Page 7-9
Outcomes and estimation	17a	For each primary and secondary outcome, results for each group, and the estimated effect size and its precision (such as 95% confidence interval)	Page 7-9, Table 3 and 4, Figure 2 and Suppl Figs 1-4
	17b	For binary outcomes, presentation of both absolute and relative effect sizes is recommended	Page 7-9
Ancillary analyses	18	Results of any other analyses performed, including subgroup analyses and adjusted analyses, distinguishing pre-specified from exploratory	Page 7-9, Table 3 and 4, Figure 2 and Suppl Figs 1-4
Harms	19	All important harms or unintended effects in each group (for specific guidance see CONSORT for harms)	Page 9 +Table 4
**Discussion**			
Limitations	20	Trial limitations, addressing sources of potential bias, imprecision, and, if relevant, multiplicity of analyses	Page 9-11
Generalisability	21	Generalisability (external validity, applicability) of the trial findings	Page 9-11
Interpretation	22	Interpretation consistent with results, balancing benefits and harms, and considering other relevant evidence	Page 9-11

**Other information**			
Registration	23	Registration number and name of trial registry	Pages 2 + 5
Protocol	24	Where the full trial protocol can be accessed, if available	N/A
Funding	25	Sources of funding and other support (such as supply of drugs), role of funders	Page 11

*We strongly recommend reading this statement in conjunction with the CONSORT 2010 Explanation and Elaboration for important clarifications on all the items. If relevant, we also recommend reading CONSORT extensions for cluster randomised trials, non-inferiority and equivalence trials, non-pharmacological treatments, herbal interventions, and pragmatic trials. Additional extensions are forthcoming: for those and for up to date references relevant to this checklist, see www.consort-statement.org.
